# The role of lipocalin-2 in lung homeostasis, inflammation response, and recovery following organic dust exposure

**DOI:** 10.1371/journal.pone.0359338

**Published:** 2026-11-03

**Authors:** Stephanie Guardado, Daniel Ojeda-Juárez, Arzu Ulu, Stefanie Sveiven, Edward C. Dominguez, Sang Yong Kim, Shaiva Patel, Marcus Kaul, Tara M. Nordgren

**Affiliations:** 1 Division of Biomedical Sciences, School of Medicine, University of California Riverside, Riverside, California, United States of America; 2 Department of Pediatrics, School of Medicine, University of Nebraska Medical Center, Omaha, Nebraska, United States of America; Novartis Institutes for BioMedical Research, UNITED STATES OF AMERICA

## Abstract

Agricultural industry workers are frequently exposed to harmful dust particles and consequently have a higher risk of developing chronic lung inflammatory conditions such as chronic obstructive pulmonary disease (COPD). Lipocalin-2 (LCN-2) is an innate immune protein with paradoxical roles in both pro- and anti-inflammatory responses at mucosal sites; however, its role in the lung during homeostatic and inflammatory states, such as those induced by inhaled dusts, remain unknown. Here, we investigated the role of LCN-2 in a murine model of organic dust exposure-induced lung inflammation using wildtype C57Bl/6 (WT) and LCN-2 knockout (KO) mice exposed repetitively to extracts of dusts (DE) collected from swine confinement facilities. Repetitive DE exposure consisted of fifteen days of intranasal instillations with and without a three-day recovery period. Through these investigations, we identified an important function of LCN-2 in regulating tissue homeostasis and recovery following inflammatory injury in the lung. Additionally, compartmentalized effects on T cell and macrophage levels were observed in bronchoalveolar lavage fluid (BALF) vs. lung tissue in DE-exposed LCN-2 KO mice versus WT mice. Ablation of LCN-2 resulted in deficits in resolving histopathological markers of inflammation, and decreased levels of IL-10 after DE. Exogenous IL-10 administration partially rescued indicators of inflammation in LCN-2 KO mice after DE exposure. Taken together, these investigations highlight a novel role for LCN-2 in regulating lung homeostasis, inflammation response, and repair following organic dust-induced inflammation that may be mediated, at least in part, by IL-10.

## Introduction

Agricultural industry workers are recognized by the Centers for Disease Control and Prevention (CDC) as individuals who are at a higher risk of developing inflammatory lung diseases such as asthma and chronic obstructive pulmonary disease (COPD) [1,2]. Workers in these industries are frequently exposed to harmful particulate matter (dusts) and vapors [1,2]. Prolonged exposure to these respirable dusts, and particularly dusts generated in concentrated animal feeding operations (CAFOs), including those housing swine, can cause severe inflammation leading to debilitating chronic lung diseases [1,2]. Since COPD is a chronic condition that causes limited airflow in the lungs, other complications such as cardiovascular diseases can arise and are common causes of death in patients with the disease. Currently, COPD is the third leading cause of death worldwide, and although preventable, there is no known cure. Current therapeutic options treat disease symptoms but not the cause of underlying disease and are not curative, thus providing no aid in reversing the decline of lung function associated with COPD [3]. As a result, it is crucial to find a treatment approach that will promote and utilize processes that promote lung tissue repair.

Lipocalin-2 (LCN-2) is an innate immune protein with paradoxical roles in both pro-inflammatory and anti-inflammatory processes that are significantly elevated at mucosal sites under inflammatory conditions [4–6] (Fig 1). One well-documented role of LCN-2 in innate immunity is the inhibition of bacterial growth via its ability to sequester iron and consequently limit iron availability necessary for bacterial proliferation [7]. Additionally, LCN-2 promotes inflammation by increasing MyD88 and NF-kB activities and by recruiting neutrophils to sites of inflammation [8]. The anti-inflammatory roles of LCN-2 include its capacity to skew towards an M2-like (alternatively activated) macrophage polarization [5] and the enhancement of regulatory T cells [4]. Although LCN-2 has such immune protective effects at mucosal sites of various tissues, its role remains unclear in organic dust-induced lung inflammation (such as dusts found in agricultural environments). Most studies examining the role of LCN-2 in lung inflammation have been performed using models of bacterial infection. Organic dusts are composed of bacterial particulates; however, these particulates are not often associated with active bacterial infections [9]. LCN-2 has been shown to be elevated in the lungs in response to inflammatory insults, including during exposure to dust extracts (DE) [10–13]. In particular, previous findings from our lab using DE for a 24-week chronic study assessing the impact of DE on pro-carcinogenic pathways identified LCN-2 as one of the most differentially upregulated genes using a NanoString Cancer panel [10], warranting additional investigation into the role of LCN-2 in the lung inflammatory response to DE.

**Fig 1:**
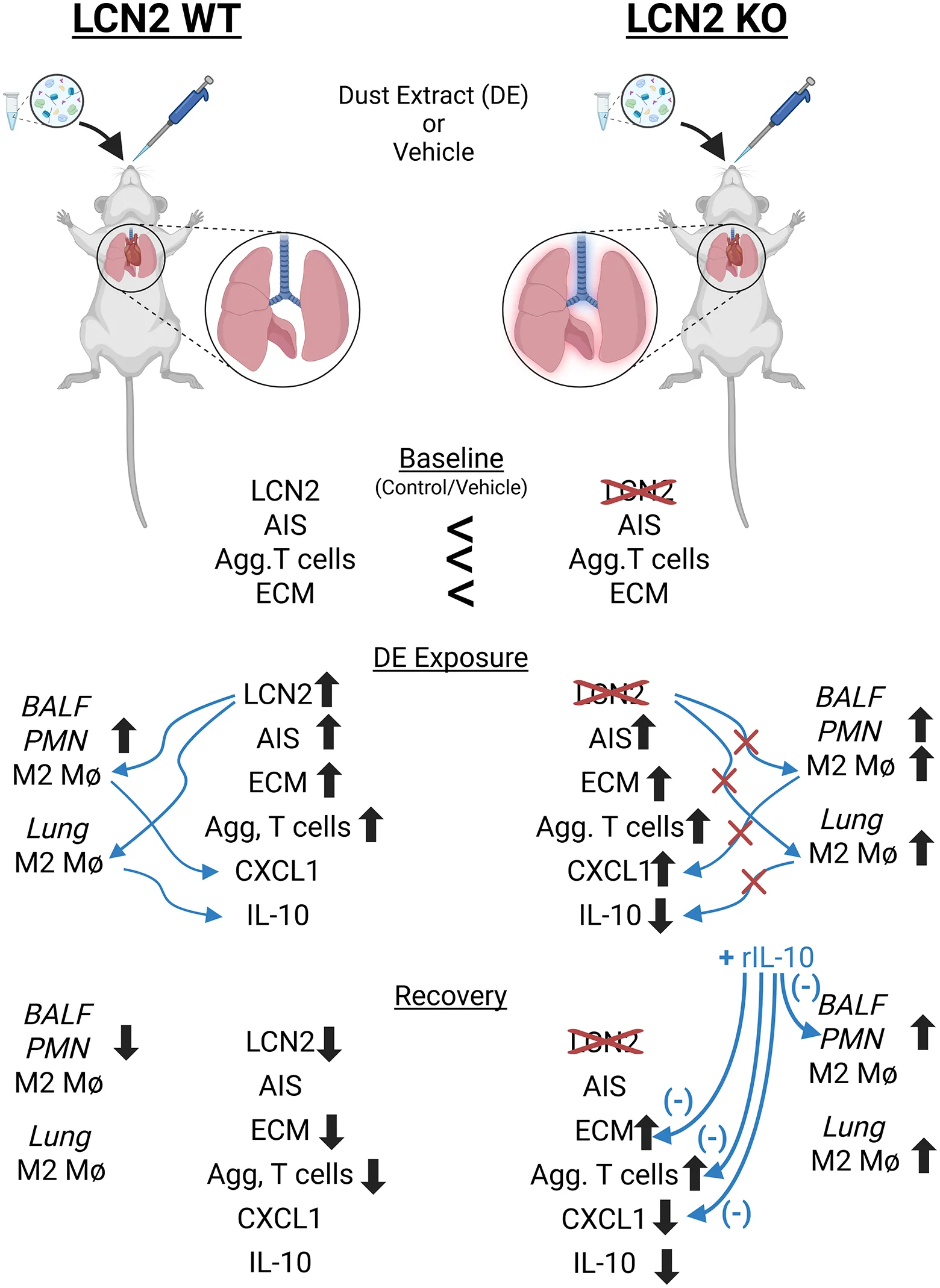
Proposed Effects mediated by LCN-2 and IL-10 in Lung Inflammation Induced by Dust Extract (DE). Exposure to DE is associated with pro- and anti-inflammatory effects. Notably, compared to wild-type control (LCN2 WT) genetic LCN-2 deficiency (LCN2 KO) is at baseline (Control/Vehicle) associated with relatively higher alveolar inflammatory score (AIS) as well as more T cell aggregates (Aggr. T cells) and deposition of extracellular matrix (ECM). Exposure to DE leads to increased LCN-2, pro-inflammatory neutrophil (PMN) infiltration, and lung tissue pathology (denoted with black solid upward arrows). The pro-inflammatory chemokine CXCL1 is primarily increased in the absence of LCN-2 and a decrease of anti-inflammatory IL-10 is only observed in LCN2 KO. During the recovery phase, ECM deposits and T cells aggregates remain unresolved and CXCL1 levels elevated in the absence of IL-10. Treatment with recombinant murine IL-10 (rIL-10) reduces levels of PMN, ECM deposits, and T cell aggregates and facilitates decline of CXCL-1. Thus LCN-2 appears to be critical for inducing IL-10 at levels that permit full recovery. Effects of LCN-2 in regulation of inflammation and IL-10 are denoted by blue arrows. Created in BioRender. Kaul, M. (2026) https://BioRender.com/o0vhxvd.

To further explore the role of LCN-2 in modulating lung tissue responses to organic dusts, we performed repetitive DE exposures in WT and LCN-2 KO mice. To evaluate the role of LCN-2 in lung tissue injury and repair, these studies included tissue collection at five hours after the last instillation as well as during the recovery period at three days following the last instillation. Lack of LCN-2 resulted in persistence of lymphoid aggregates in recovery, as well as decreased levels of IL-10 after DE exposure. Given the recognized roles of IL-10 in tissue repair, these results prompted us to investigate the crosstalk between LCN-2 and IL-10 following DE exposure. To this end, we performed repetitive exposure to DE in LCN-2 KO mice followed by IL-10 administration during the recovery period. Our results revealed that LCN-2 KO mice had deficits in recovery and that IL-10 administration could partially rescue these deficits following DE exposure. These findings suggest a protective role of LCN-2 in the lung in response to DE and provide evidence for LCN-2 as an immune regulator in the lung.

## Materials and methods

### Preparation of DE and IL-10

The DE used for these experiments was derived from settled surface dusts collected from swine concentrated animal feeding operations (CAFOs) housing ~500−700 animals, as previously described [14–16]. The same stock of collected surface dusts were used for all experiments reported herein. The extracts were prepared by dissolving 1 g of dust in 10 ml of Hank’s balanced salt solution (Biofluids, Rockville, MD, USA) without calcium. After vortexing, the mixture was incubated at room temperature for 1 hour. The mixture was then centrifuged for 10 minutes, and the supernatant fraction was recovered. The mixture was then centrifuged once more, and the final supernatant fraction, 100% DE, was filter-sterilized, aliquoted and stored at -80^o^C until used. For all studies, the 100% DE was further diluted to 12.5% with saline. Recombinant IL-10 (Biolegend, San Diego, CA USA; Catalog #575804) was diluted to a concentration of 0.02 µg/µl with 1X sterile PBS.

### Animal care and housing

Male 6- to 8-week-old C57BL/6J mice were obtained from Jackson Laboratories (Bar Harbor, Maine) and placed in group housing cages under pathogen-free conditions and used as controls for experiments at the age of 8–13 weeks old. Male LCN-2 knockout (KO) mice were obtained from breeding male and female LCN-2 homozygous KO mice (B6.129P2-*Lcn2tm1Aade*/AkiJ), and used for experiments at the age of 8–13 weeks old. These mice were backcrossed for at least 10 generations to C57BL/6J mice, and upon arrival at The Jackson Laboratory, were bred with C57BlL/6J for at least one generation, making C57BL/6J mice appropriate as a wild-type comparator (See development details for The Jackson Laboratory Strain #:024630). Mice were fed *ad libitum* standard mouse chow diet and water. All experimental procedures and protocols involving animals were performed by trained personnel in accordance with NIH guidelines and approved by the University of California, Riverside, Institutional Animal Care and Use Committee and the study follows the ARRIVE guidelines. Mouse health and diet were supervised by the University of California, Riverside campus veterinarian. Animals were monitored daily throughout the duration of the studies for signs of distress that would require intervening (humane endpoint), including severe weight loss (>15%), lack of eating/drinking, respiratory distress, hunching, and other signs of pain or discomfort. No animals in these studies experienced unexpected mortality or required euthanasia due to any of these criteria. Beyond the use of isoflurane during the intranasal instillations, no interventions for minimization of suffering/distress, including the use of analgesics or special housing conditions were required. All animals (N = 106) were euthanized at their expected time points via isoflurane overdose followed by cervical dislocation and thoracotomy as secondary measures of euthanasia.

### *In vivo* DE exposure and sample collection

WT and LCN-2 KO mice were repetitively exposed to DE prepared from dusts collected from swine confinement facilities or saline vehicle control. An aqueous solution of DE was prepared as described above and published previously [17–19] and administered to mice as a 50 µl intranasal instillation (IN) of 12.5% DE or saline under light isoflurane anesthesia [18]. Male mice of 8–13 weeks old were given IN in the mornings for 15 weekdays, with instillations provided for 5 consecutive days followed by 2 days rest over a 3 week period (Fig 2). For this work, we evaluated two time points after repetitive DE exposure: 1) five hours after last IN (repetitive exposure without recovery period), and 2) 72 hours after last IN (repetitive exposure with recovery). For studies that included IL-10 administration, 1 µg of recombinant mouse IL-10 per 25 g mouse body weight was administered to LCN-2 KO animals on the day of the final DE IN and at 48 hours post final DE IN. Animals were euthanized with isoflurane and collection of bronchoalveolar lavage fluid (BALF) and lung tissue was performed as described below. For BALF collection, a cannula was first inserted into the trachea and secured by tying a knot with a suture around the trachea. A syringe was used to rinse/wash the whole lung with 1 ml of sterile 1X PBS, which was collected in a separate tube to prevent over-dilution in subsequent enzyme-linked immunoassays (ELISAs). The lungs were washed two additional times with 1 ml of 1X PBS and these washes were combined in a second tube for downstream cellular analyses. After BALF collection, the left lung lobe was tied off, excised and frozen in RNAlater to be used for NanoString analyses. Alternatively, in some samples lung lobes were excised and used for the preparation of single cell suspensions for use in flow cytometry (detailed below). The right lung lobes were inflated with 0.5 ml of 10% formalin and hung with a constant pressure of 20 cm H_2_O for 24 hours while submerged in formalin for optimal lung morphology preservation (N = 3–8 mice per experimental group).

**Fig 2:**
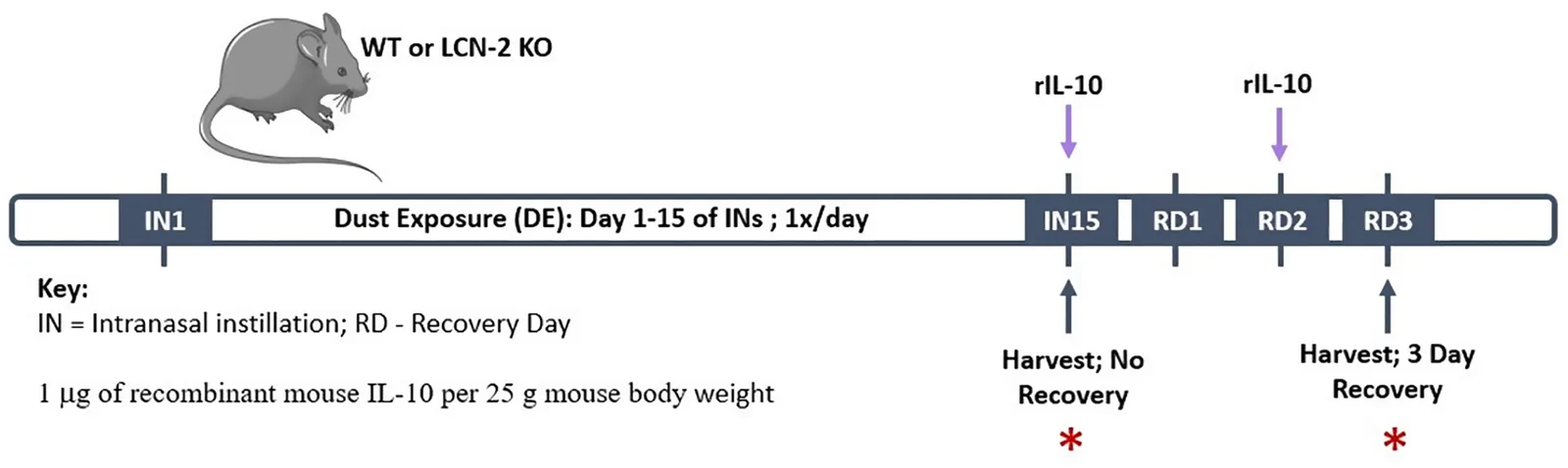
Murine Model Timeline of DE Exposures, Harvests, and/or rIL-10 Administration. Mice received intranasal instillations (IN) of DE for 15 consecutive weekdays. For experiments with rIL-10 administration, 1 µg/25 g dose was given to mice on the last IN and on the 2^nd^ recovery day. Harvests were performed 5 hours or 3 days post last IN. Portions of this figure were prepared using images from Servier Medical Art. Servier Medical Art by Servier is licensed under a Creative Commons Attribution 3.0 Unported License.

### Staining for differential cell counts using BALF and lung tissue

Cell pellets from the three BALF washes described above were combined per mouse and used for cytospins and flow cytometry. Both BALF tubes were centrifuged for 5 minutes at 500 rcf and the supernatant fraction from the tube containing the first 1 mL wash was collected and used for cytokine/mediator detection by ELISA. Cells from both tubes (i.e., all three washes) were pooled together. Red blood cells were lysed in ACK lysing buffer (Thermo Fisher Scientific, Waltham, MA, USA; Catalog #8485741) for 5 minutes at room temperature and the remaining cells were resuspended in 1X PBS. A hemocytometer was used to enumerate cells in BALF samples and cytospins were made using an Epredia Cytospin Centrifuge (VWR, Radnor, PA, USA; Catalog# 89370−864). Cytospins were stained with a Diff-Quik protocol (Siemens Diff-Quik Stain Set; Newark, Delaware) for cell differential analyses. Percent macrophage and neutrophil populations were enumerated per 300 cells per mouse. Additionally, processed BALF and lung cell suspensions were stained using a panel of antibodies for flow cytometry analyses of various cell populations, as described below.

### LCN-2 and cytokine measurements in BALF

Collected BALF supernatant fractions described above were used for detecting mouse LCN-2 (Catalog #DY1857), IL-10 (Catalog #DY417), and CXCL1 (Catalog #DY453) levels using R&D Systems (Minneapolis, MN, USA) Duoset ELISA kits following the manufacturer’s instructions. A VarioSkan microplate reader (Thermo Scientific, VL0000D0) was used to read ELISA plates at the wavelengths recommended by the manufacturer (R&D Systems).

### Flow cytometry

Collected BALF cells or single cell suspensions from lung tissue were prepared for flow cytometric assessment of immune cell populations. For lung tissue samples, single cells suspensions from excised lung lobes were minced by scalpel and then digested in a solution of 5% fetal bovine serum (FBS), 1 mg/mL Roche’s collagenase A, and 0.5 mg/mL Sigma DNase I in Hank’s balanced salt solution for 1 hour in a shaking incubator at 37.5ºC. With 15 minutes remaining in the incubation period, lung digests were further homogenized using an 18-gauge needle (10 times) and returned for the final 15 minutes of incubation. The single cell suspensions were filtered with 70 µm cell strainers and pelleted before red blood cell (RBC) lysis (performed according to manufacturer’s proptocl [BioLegend]). The flow cytometry panel consisted of the following antibodies purchased from Biolegend (San Diego, CA, USA), Thermo Fisher Scientific (Waltham, MA, USA), BD Biosciences (San Jose, CA, USA), and Bio-Rad (Hercules, CA, USA): MERTK-FITC (Catalog #151503), CD11b-PE (Catalog #12-0112-82), SiglecF-PE-Texas Red (Catalog #562757), CD3-PerCP-Cy5.5 (Catalog #45-0031-82), Ly6C-PE-Cy7 (Catalog #25-5932-82), CD206-AF647 (Catalog #MCA2235A647), MHC II-AF700 (Catalog #107621), B220-A780 (Catalog #47-0452-82), Ly6G-BV510 (Catalog #127633), Cd11c-QDOT605 (Catalog #117334), and CD86-QDOT655 (Catalog #50402970). Briefly, cells were stained with Zombie Violet (Biolegend, San Diego, CA, USA; Catalog #423114) LIVE/DEAD stain at conditions recommended by the manufacturer, blocked with 1:50 Fc blocking buffer (BD Biosciences, San Jose, CA, USA; Catalog #553142), and stained with a master mix containing all antibodies at a final dilution of 1:100. Compensation was performed using the Thermo Fisher Scientific ABC Total Antibody Compensation Bead Kit (Catalog #A10497). Samples were analyzed in a BD Biosciences LSRII Flow Cytometer and resultant data were analyzed using the FlowJo software (Ashland, OR, USA). Gating strategy is provided in S1 Fig.

### Scoring of lung pathology

All formalin-fixed lungs used for histological assessment were first subjected to BALF collections, enabling complementary endpoints from the same animal. After fixation (described above), lungs were placed in 70% ethanol and shipped to the University of California Irvine Pathology Research Services Core for paraffin embedding and sectioning. Lung tissue sections were subsequently stained with hematoxylin and eosin or with the Richard Allan Scientific Masson’s Trichrome Kit (Thermo Fisher Scientific, Waltham, MA, USA; Catalog #22-110-648) for extracellular matrix (ECM) deposition scoring. Observers were blinded to exposure and treatment conditions for each slide. Evaluation of lung inflammation was performed by scoring H&E-stained slides for alveolar inflammation, bronchiolar inflammation, epithelial hyperplasia, and lymphoid aggregates, and Masson’s Trichrome-stained slides were used to quantify pro-fibrotic changes. Scoring for lymphoid aggregates, alveolar and bronchiolar inflammation was done by scanning each slide at 20X and 40X followed by enumerating cells at 40X for 10 images per mouse total. Each slide was scored for the degree of inflammation using scores (0–3) with higher scores representing the most severe inflammation. For epithelial hyperplasia and lung pro-fibrotic changes, each slide was scored for the degree of inflammatory/fibrotic pathology using scores (0–4) with higher scores increasing epithelial thickness and collagen deposition (marked by blue coloration), as previously described [10,14,15].

### NanoString gene expression

Mouse NanoString Fibrosis V2 panels (Catalog #115000388; NanoString Technologies, Seattle, WA, USA) were used for quantification of 778 RNA transcripts using an nCounter Sprint Profiler. Three mouse lungs were used from independent experiments for each experimental group. Upon harvest and proper storage as previously described above, mouse lungs were thawed for RNA extraction. Lungs were rinsed with PBS followed by homogenization in 1 ml Trizol by using a 7 cm polypropylene pellet pestle in a microcentrifuge tube. A Purelink RNA Mini kit (Catalog #12183025; Invitrogen, Carlsbad, CA, USA) was used for the RNA extraction, which was performed per the manufacturer’s instructions. RNA integrity number (RIN) was acquired from the UC Riverside Institute for Integrative Genome Biology Core Facility by using an Agilent Bioanalyzer 2100 (Agilent, Santa Clara, CA, USA). All samples used had a RIN of 7–9. A codeset probe provided by the kit was hybridized for 18 hours with 50 ng RNA. Samples were brought up to volume with nuclease-free water and 32 µl were loaded onto an nCounter Sprint Cartridge (Catalog #100078; NanoString Technologies, Seattle, WA, USA). Each study cohort consisted of 12 samples, with one of three cohorts loaded per 12-sample cartridge: 1) repetitive exposure without recovery or IL-10 administration; 2) repetitive exposure with recovery but no IL-10 administration; and 3) repetitive exposure with recovery and IL-10 administration. All samples passed the NanoString system quality control (QC) test. Data were analyzed using nSolver 4.0 and by performing a NanoString Advanced analysis. The following housekeeping genes showing the strongest correlation with each other were used for normalization of gene expression: *Acad9, Cnot10, Mtmr14, Nol7, Nubp1, Pgk1, Ppia, and Rplp0*. The agglomerative analysis on nSolver was used to generate heatmaps. Per NanoString’s recommendation, 50 transcript counts was taken as the “count threshold”. The 20 most upregulated genes among all treatments by P-value were identified by differential expression analysis. STRING database (https://string-db.org) was used to explore protein-protein interactions among differentially regulated genes from NanoString that were statistically significant based on the unadjusted p-values [20]. Nanostring data can be found in the NCBI Gene Expression Omnibus Repository: https://www.ncbi.nlm.nih.gov/geo/query/acc.cgi?acc=GSE254514.

### RNAscope

RNAscope® experiments were performed using the formalin-fixed paraffin-embedded (FFPE) tissue protocol for the RNAscope® Multiplex Fluorescent Reagent Kit v2 Assay (Document Number: 323100-USM). FFPE tissues were prepared as previously described and sectioned at 5 µm. Three mouse lungs were used per condition from independent experiments. The following probes, RNAscope® 3-plex Negative Control (Catalog #320871), RNAscope® 3-plex Positive Control (Catalog #320881), RNAscope® LCN-2 (Catalog #313971), and RNAscope® IL-10 (Catalog #317261-C2) were purchased from Advanced Cell Diagnostics, Inc. (Newark, CA, USA). Opal 570 (Catalog #FP14880001KT; 1:750) and Opal 690 (Catalog # FP14970001KT; 1:750) dyes from Akoya Biosciences (Marlborough, MA, USA) were used to visualize hybridized probes. Upon completion of the RNAscope® staining protocol, tissues were blocked with 10% goat serum for 90 minutes and incubated with 1:150 diluted Ym-1 (Catalog #60130; Stem Cell Technologies, Cambridge, MA, USA) in 5% goat serum at 4^o^C overnight. The following day, tissues were incubated with 1:500 of goat anti-rabbit-488 antibody for 1 hour at RT, then stained with Hoechst 33342 dye (Catalog # B2261; Sigma) for 5 minutes at RT. Slides were mounted with Vectashield (Catalog #H1000; Vector Laboratories, Burlingame, CA 94010, USA), cured overnight, and stored at 4^o^C in the dark until the following day. An Axiovert 200M fluorescence microscope (Zeiss) with filters for DAPI, FITC, Cy3 and Cy5 was used to acquire Z-stacks of images. The Slidebook software (Version 6, Intelligent Imaging Innovations, Denver, CO) was used to analyze the images and quantification of mRNA was performed by enumerating the sum of intensity per dots.

### Statistical analysis

GraphPad Prism software 7 (San Diego, CA, USA) was used to perform unpaired t tests, Kruskal Wallace tests, two-way ANOVA or one-way ANOVA tests for statistical analysis as appropriate. For ANOVA analyses, Tukey’s post-hoc analyses were used to assess for significant differences between groups, correcting for multiple comparisons. For nonparametric tests, multiple comparisons were corrected for by controlling the false discovery rate (FDR) via the two-stage step-up method of Benjamini, Krieger and Yekutieli. A significance cut-off for all statistical analyses set at a *P* value ≤ 0.05. GraphPad Prism Software was used to generate all graphs. In figures, each symbol represents a single animal; in figures without symbols, N are indicated.

## Results

### LCN-2 KO mice exhibit disruption of tissue homeostasis with worsened inflammatory pathology and impaired recovery in a model of repetitive organic dust exposure

To determine the role of LCN-2 in lung injury and recovery, we utilized a self-resolving mouse model of organic dust-induced inflammation. WT and LCN-2 KO animals were challenged with repetitive organic DE exposure consisting of 15 doses across 3 weeks, and tissues were collected at 5 hours or 3 days following final instillation challenge to elucidate the role of LCN-2 in the initial tissue damage and recovery phase, respectively. When assessing for LCN-2 expression in this model, we detected significant levels of LCN-2 protein in the lavage of both control (saline)- and DE-instilled animals at both 5 hours and 3 days following final instillation challenge as measured via ELISA (Fig 3A). The BALF basal levels of LCN-2 protein were high (~140,000 pg/ml), which increased to ~660,000 pg/mL in response to DE at the 5 hours post-exposure timepoint. The basal level of LCN-2 in BALF represents an approximately three-fold increase in comparison to basal levels of LCN-2 in serum (where LCN-2 was present at ~50,000 pg/ml in saline-instilled mice and ~77,000 pg/mL in DE-instilled animals; S2 Fig). We also verified *Lcn-2* transcript levels in the lung, identifying robust induction of *Lcn-2* transcript following DE exposure (Fig 3B-3D). Expression ranges varied broadly across the lung (see S3 Fig); however, we observed transcript expression primarily in the lung epithelium, with some expression in granulocytic cell populations as expected, and minimal overlap with YM1 + macrophage populations (Fig 3B). Furthermore, we assessed for histopathological markers of inflammation and injury in saline- and DE-challenged WT and LCN-2 KO animals. In the absence of DE challenge, LCN-2 KO mice exhibited significant differences in cellularity in lung tissue compared to WT mice, suggesting an important function of LCN-2 in regulating homeostasis in the lungs during basal conditions (Fig 4A). For all histopathological scores assessed (including alveolar inflammation, bronchiolar inflammation, epithelial hyperplasia, lymphoid aggregate formation (see Fig 4B for representative aggregates), and extracellular matrix [ECM] deposition), Kruskal-Wallis non-parametric testing was performed individually on the 5 hr time point and the recovery (3 day) time point groups and identified a significant main effect for each test). Within group comparisons for alveolar inflammation, epithelial hyperplasia, and extracellular matrix deposition, we observed non significant trends for elevated scores in LCN-2 KO animals vs. WT with saline treatment alone (Fig 4C,D), while this reached significance for an increased lymphoid aggregate score in saline-instilled KO animals compared to WT. DE exposure lead to significantly increased scores for all parameters regardless of genotype at the 5 hour timepoint (Fig 4C-4F). For the 3 day recovery timepoint, alveolar inflammation and epithelial hyperplasia remained elevated in both WT and KO DE-treated animals (Fig 4C,D), while lymphoid aggregate and extracellular matrix deposition scores returned to baseline in WT mice but remained significantly elevated in LCN-2 KO animals (Fig 4E,F).

**Fig 3:**
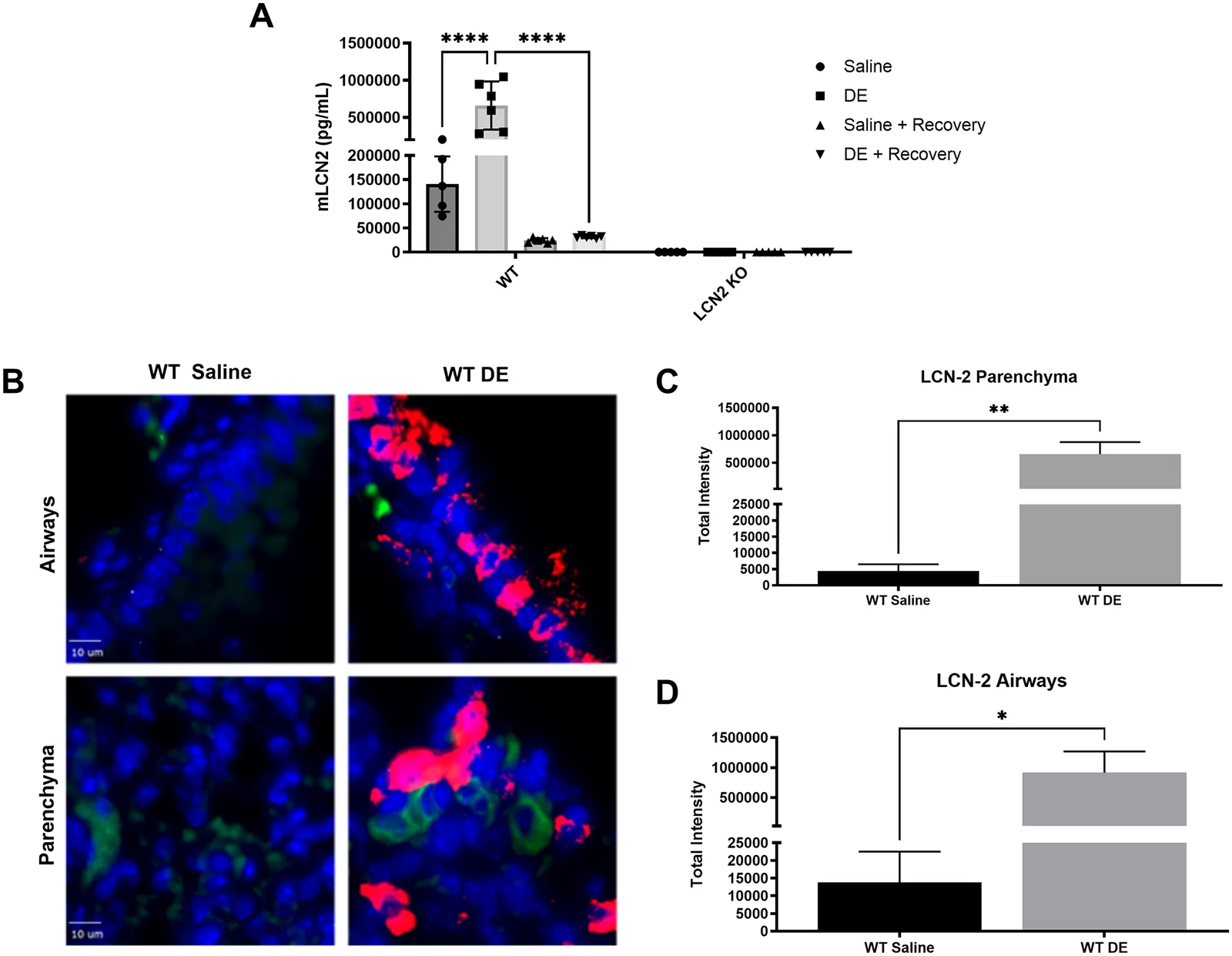
Expression of LCN-2 in the lung following DE exposure in mice. WT and LCN-2 animals were challenged with DE for 3 weeks followed by sacrifice at 5 or 72 hours following the final DE instillation to assess the impact of repetitive DE exposure on LCN-2 induction. (A) Mouse LCN-2 protein (mLCN2) in BALF of WT and LCN-2 KO mice at 5 hours and 3 days following repetitive DE exposure. (B) RNAScope images of *Lcn-2* transcript expression (Red) in lung tissues of WT saline- vs. DE-treated animals at 5 hours following final repetitive DE exposure. YM-1+ (M2-like macrophage marker) shown in green; DAPI staining is in blue. (C) Quantification of *Lcn-2* transcript levels in parenchymal lung tissue. (D) Quantification of *Lcn-2* transcript levels in Airways. *P ≤ 0.05; ** P < 0.01; **** P < 0.0001 based on 2-way ANOVA with Tukey Method for post-hoc comparisons in (A) and unpaired t tests in (C) and (D). Each symbol indicates a single animal in (A). (C) and (D) represent N = 3 mice per group, with 5 images analyzed for fluorescence intensity per animal.

**Fig 4:**
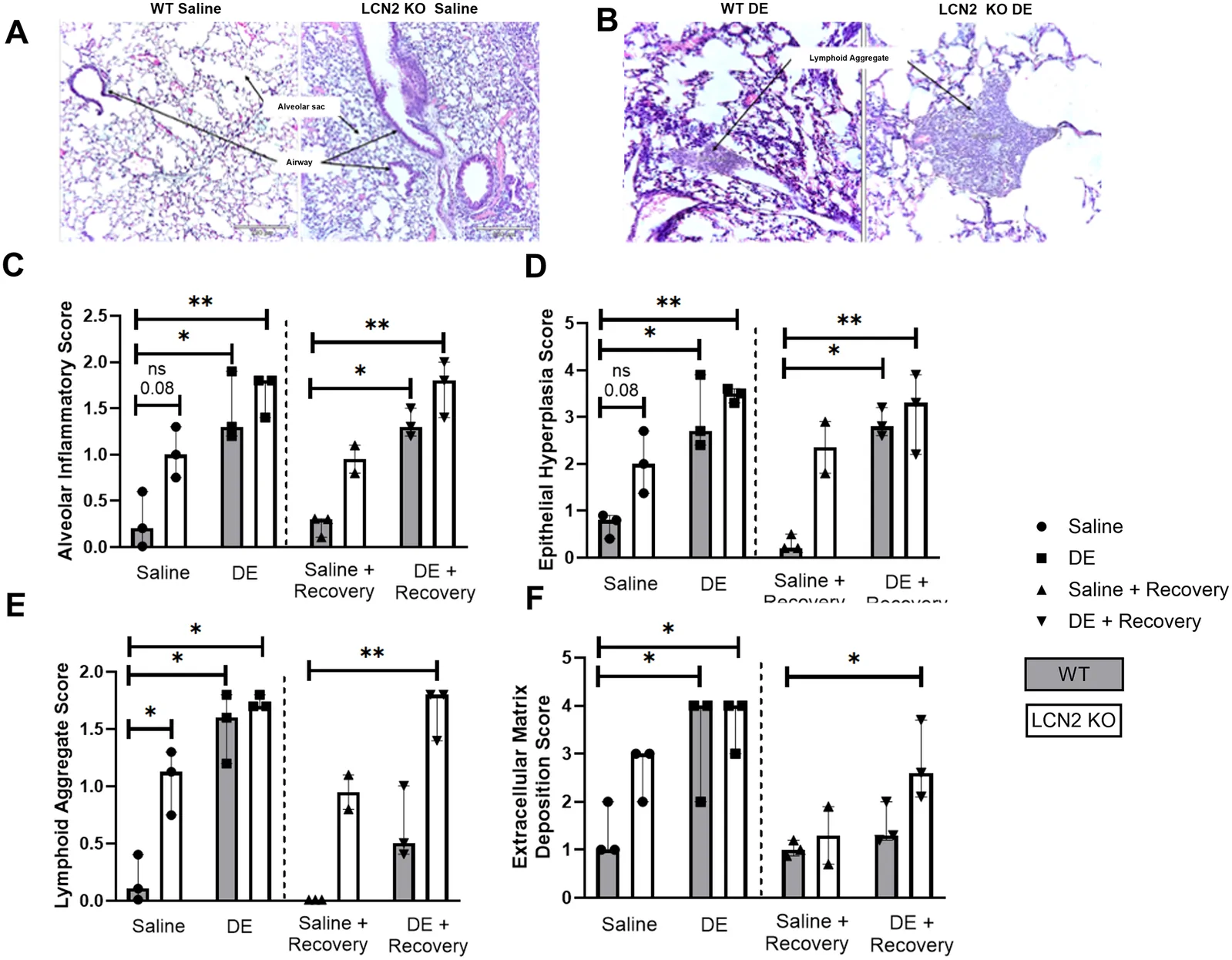
Effects of repetitive DE exposure on lung inflammatory scores at 5 hours and 3 days (recovery) following final DE challenge. WT and LCN-2 knockout animals were challenged for 3 weeks with DE, and sacrificed at 5 hours or 3 days following final DE instillation to assess impacts on lung inflammatory pathology. (A) Hematoxylin and Eosin-stained lung tissue sections of unchallenged WT vs. LCN2 KO mice; (B) Representative images of lymphoid aggregates in lung tissue sections; (C) Alveolar inflammatory score; (D) Epithelial hyperplasia score; (E) Lymphoid aggregate score; (F) Extracellular matrix deposition score. *P ≤ 0.05, *P < 0.01 based on Kruskal-Wallis test with multiple comparisons corrected via controlling FDR with a two-stage step-up method of Benjamini, Krieger and Yekutieli.

Given the significant pathology observed in LCN-2 KO mice with and without DE exposure, we next evaluated the lung immune cell populations present at 5 hours and 3 days following final DE challenge. Following DE challenge the frequencies of macrophages were decreased in the overall BALF cell population at 5 hours following exposure (Fig 5A) that coincided with a significant influx of neutrophils (CD11b+Ly6G+) in both WT and LCN-2 KO animals (Fig 5B) and this resolved by the 3-day recovery timepoint. We also observed a significant elevation in the neutrophil chemoattractant CXCL1 in DE-exposed LCN-2 KO mice at 5 hours post-exposure (Fig 5C), but this elevation did not reach significance in WT animals. Similarly, we observed a significant main effect of DE (*P* = 0.0126) with DE-treated lungs exhibiting increased percentages of neutrophils via flow cytometry, but not alveolar macrophages (Fig 5D,E).

**Fig 5:**
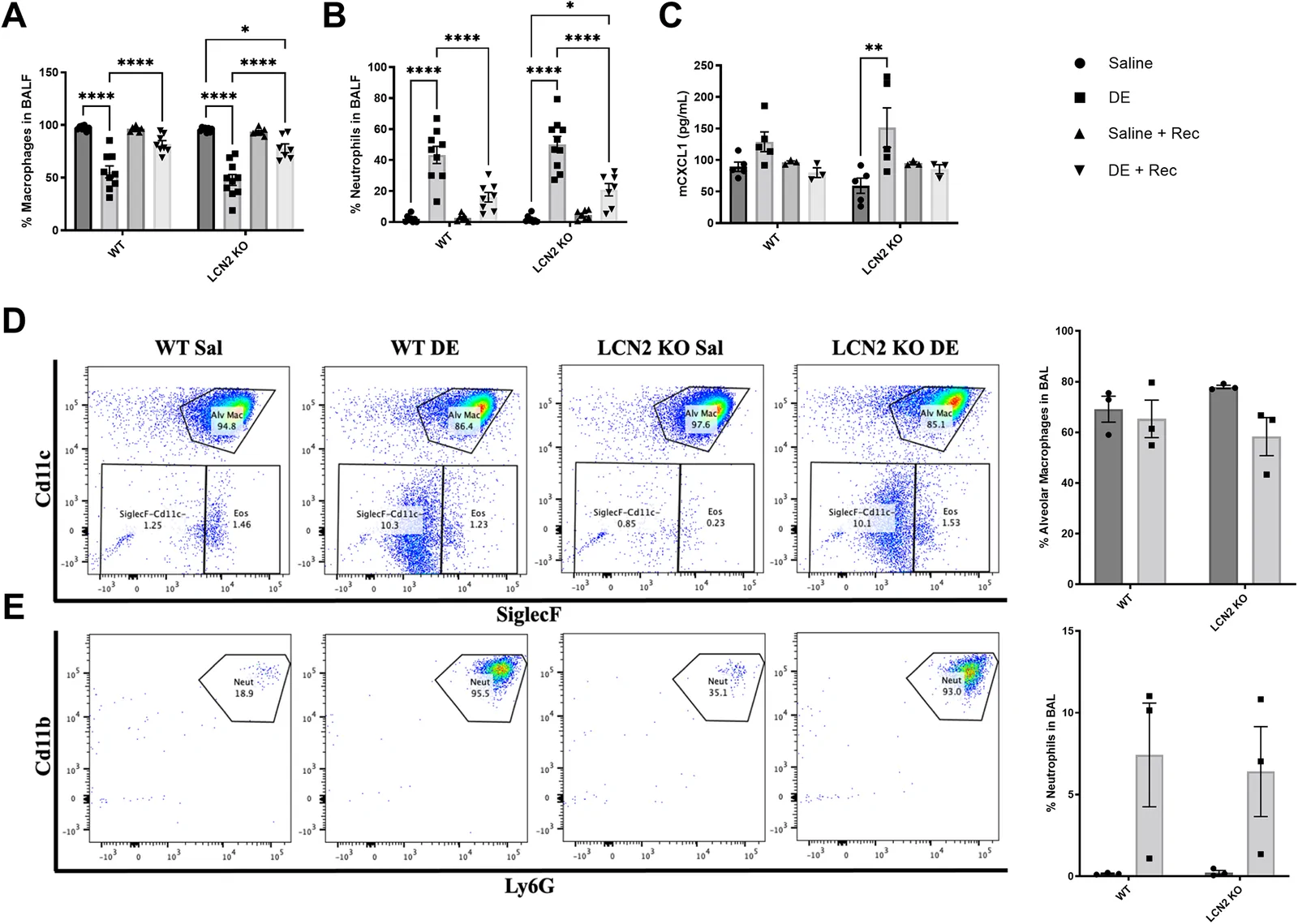
Effects of repetitive DE exposure on lung immune cell infiltration in WT and LCN-2 KO mice. In repetitively DE-challenged WT and LCN-2 KO animals, cells from BALF were enumerated using differential cell counting to identify the percent populations of macrophages (A) and neutrophils (B). Protein levels of CXCL1 were also determined via ELISA (C). Flow cytometry was used to verify BALF cell populations, including alveolar macrophages (D; Cd11c^hi^/SiglecF^hi^ [AlvMac]), and neutrophils (E; Cd11b^hi^/Ly6G^hi^ (Neut); data shown with representative gating strategies, left, and compiled results expressed as % of live cell population, right. *P ≤ 0.05; ** P < 0.01; **** P < 0.0001 based on 2-way ANOVA with Tukey Method for post-hoc comparisons. Each symbol indicates a single animal.

To determine the impact of LCN-2 on monocyte-derived macrophage polarization following DE exposure and during recovery, we used flow cytometry. We gated for alveolar and interstitial macrophages (SiglecF^high^CD11c+ alveolar and SiglecF^low^CD11c+ interstitial macrophages) to examine M2-like polarization among these macrophages, identified as CD86^low^CD206^hi^ cells. Here, we observed no significant differences in BALF populations of CD86^low^CD206^hi^ macrophages (Fig 6A; *P* = 0.1535). However, in both alveolar and interstation macrophages from lung homogenates, M2-like CD86^low^CD206^hi^ populations were significantly elevated in the DE-treated LCN-2 KO mice in comparison to their saline controls and to WT mice (Fig 6B, C), where no elevations in these populations were observed. Furthermore, T cell levels were elevated in the BALF of DE-treated LCN-2 KO mice in comparison to their saline controls, while this difference was not found in WT animals (Fig 6D), nor in lung tissue samples (Fig 6E).

**Fig 6:**
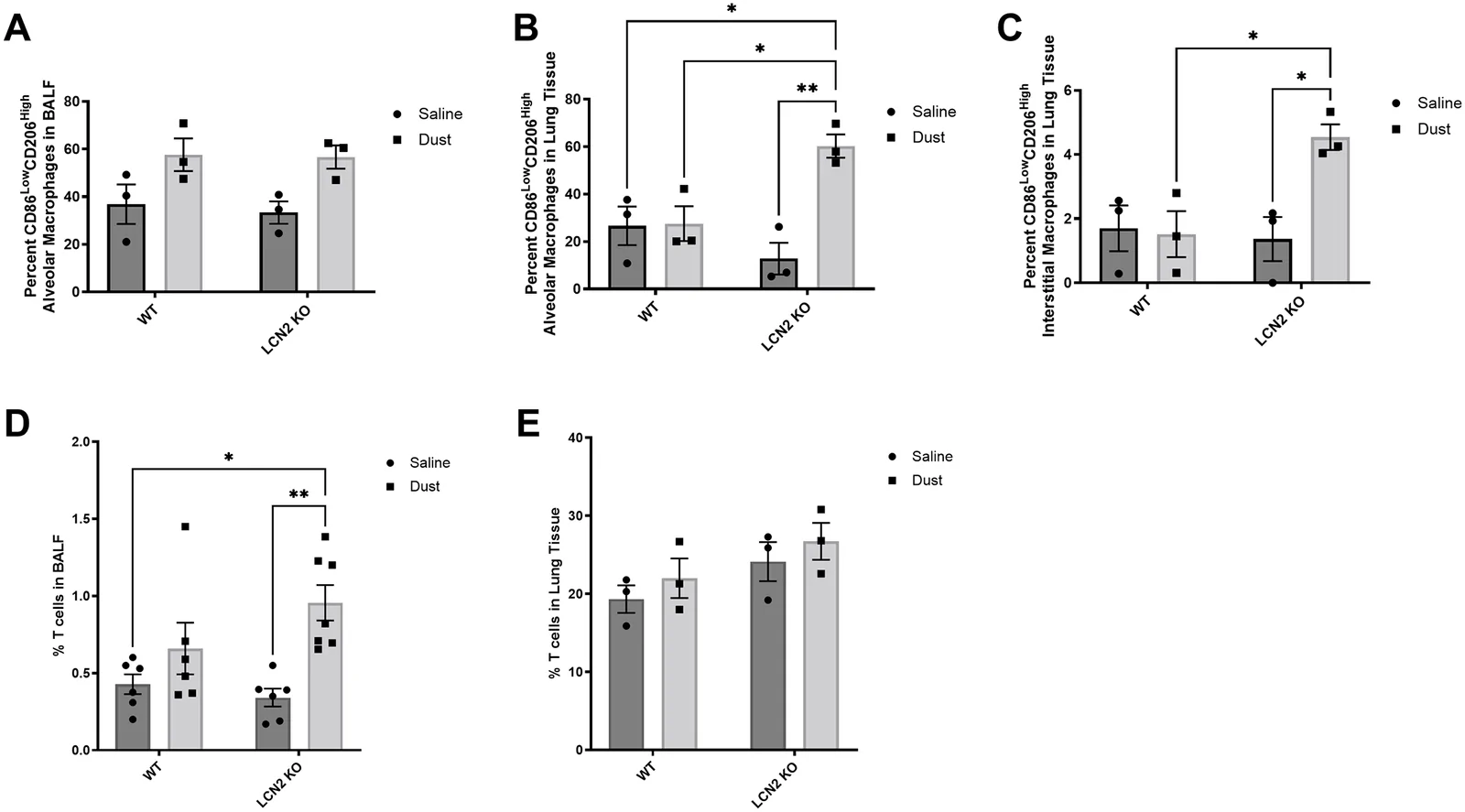
Effects of repetitive DE exposure on BALF versus lung homogenate macrophage populations in WT and LCN-2 KO mice. Flow cytometry was used to quantify CD86^low^CD206^+^ alveolar and interstitial macrophages in BALF (A) and lung tissue homogenates (dissociated cell suspensions; C, D). After gating for alveolar and interstitial macrophages amongst live cells, CD86 was used to identify M1-like (Classically activated) and CD206 for M2-like (alternatively activated) macrophages. M2-like macrophages were identified as CD86^low^CD206^+^ cells. T cell levels were also assessed via flow cytometry in BALF (B) and lung homogenates (E). *P ≤ 0.05; ** P < 0.01; based on 2-way ANOVA with Tukey Method for post-hoc comparisons. Each symbol indicates a single animal.

To further explore the signaling pathways and specific genes altered by LCN-2 ablation, we performed NanoString gene expression analysis. The log2 fold change differential expression data observed between experimental groups are summarized in Fig 7. The top 20 differentially regulated genes identified based on DE exposure, genotype, and recovery time point (5 hours vs. 3 days) are shown in Fig 7A, 7B, and 7C, respectively, all compared to a baseline of WT Saline controls. Comparison based on WT vs. LCN-2 genotype grouping identified 36 total differentially regulated gene transcripts, and interactions of all 36 genes are visualized in Fig 7D. Here, String-db.org was utilized to explore the network of genes, identifying a protein-protein interaction (PPI) enrichment P-value of 1.07e-06, and also identifying two associated Reactome pathways for “Immunoregulatory interactions between a lymphoid and a non-lymphoid cell” (MMU-198933; false discovery rate of 0.0457) and “Extracellular matrix organization: (MMU-1474244; false discovery rate of 0.0461). These data corroborate the significant main effects and/or DE-associated differences in ECM deposition and lymphoid aggregates observed in LCN-2 KO mice based on histopathological analysis, suggesting that absence of LCN-2 is associated with abrogation in resolution/repair mechanisms and/or aberrant fibroproliferative remodeling (Fig 4).

**Fig 7:**
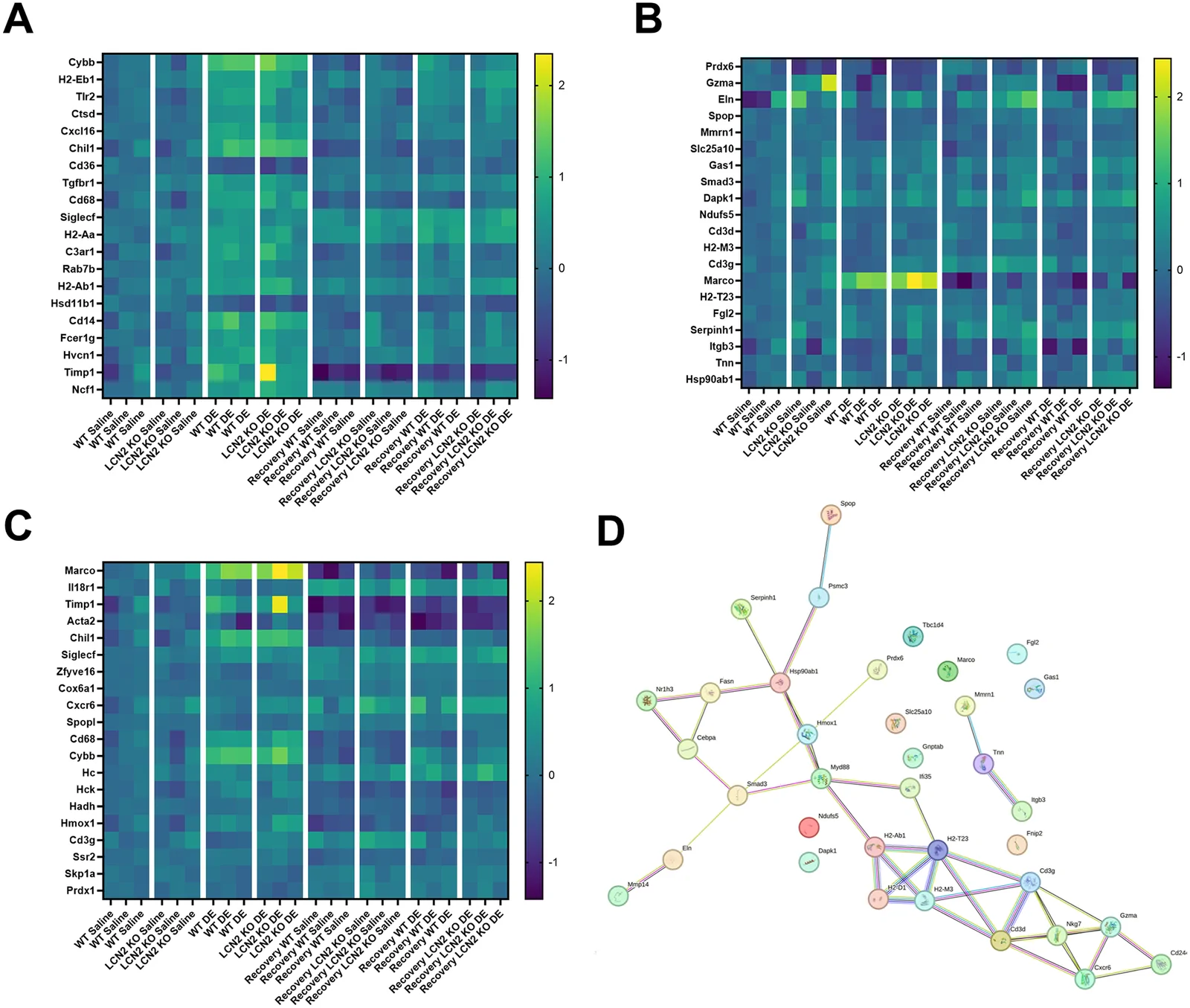
NanoString analysis of differentially regulated gene transcripts in mouse lungs of DE-exposed WT and LCN-2 KO mice with and without a recovery period. Nanostring gene expression profiling was performed to identify differentially regulated gene transcripts in DE- or saline-exposed WT and LCN-2 KO animals with and without a 3-day recovery following repetitive DE exposure. All heatmaps portray log(2) fold changes compared to a baseline of WT Saline controls. (A) Top 20 differentially regulated genes by P-value based on exposure. (B) Top 20 differentially regulated genes by P-value based on genotype. (C) Top 20 differentially regulated genes by P-value based on recovery period (5 hours vs. 3 days). (D) String-db cluster map of gene interactions amongst the 36 differentially regulated gene transcripts based on genotype. N = 3 for each experimental group in NanoString analyses.

### Recombinant IL-10 therapy partially rescues LCN-2 KO-associated repair deficits following DE exposure

During bacterial infection, LCN-2 is induced by IL-10 to skew macrophages towards an anti-inflammatory M2-like phenotype, and consequently worsens outcomes by impairing bacterial clearance [5]. Importantly, DE contains immunogens consisting of bacterial components and induces IL-10 expression [9,21]. In the context of DE exposure, the IL-10/LCN-2 axis may be essential for the resolution of inflammation and recovery. We hypothesized that LCN-2 KO-associated alterations in the IL-10 signaling pathway could account for the observed persistence in inflammatory/pro-fibrotic remodeling during recovery following DE exposure. To test this, we measured the levels of IL-10 in BALF in WT and LCN-2 KO mice after DE exposure, where we observed a decrease in IL-10 levels in LCN-2 KO mice after DE exposure, as compared to WT-DE-exposed mice (Fig 8A).

**Fig 8:**
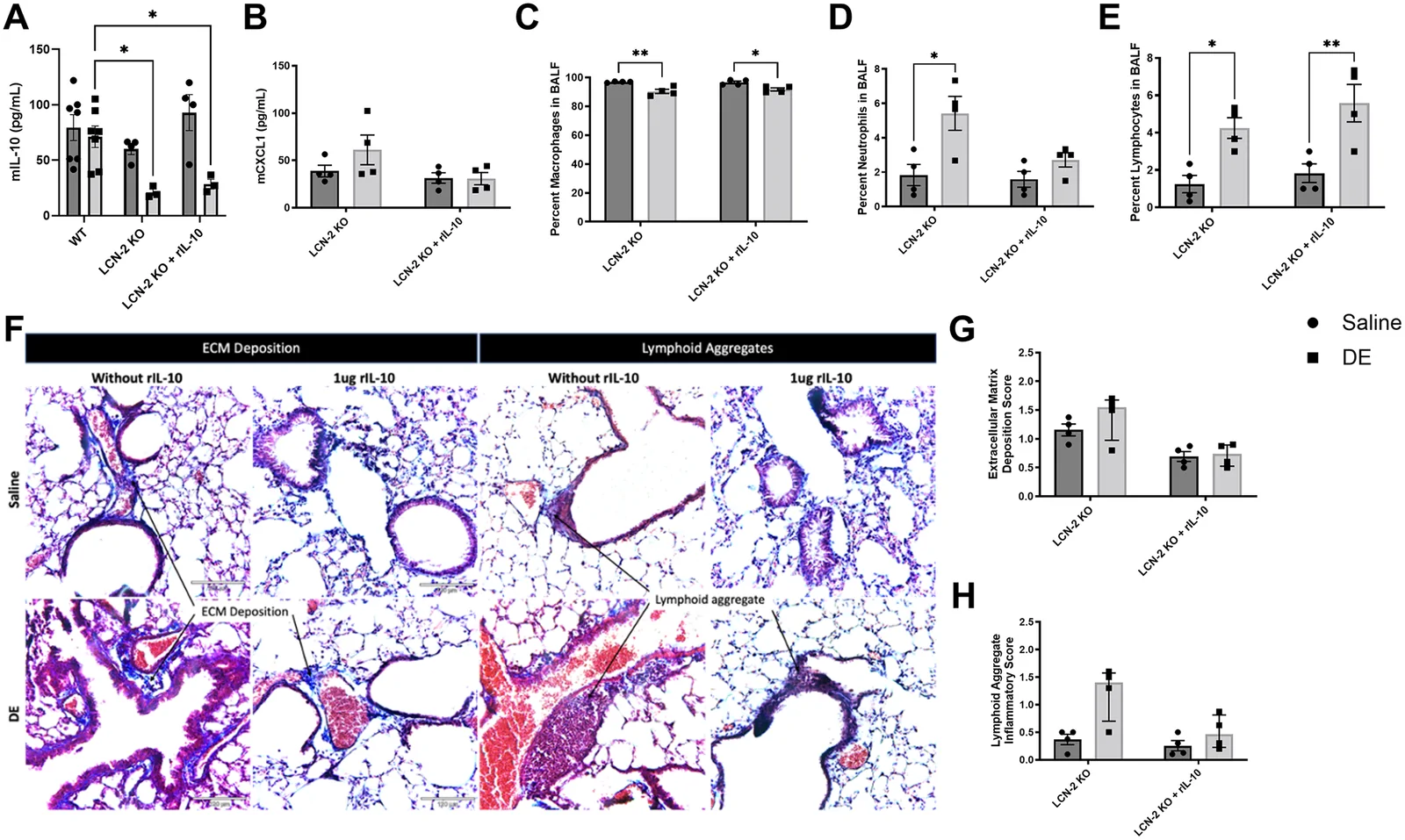
Effects of rIL-10 administration on lung recovery at 3 days following repetitive DE exposure in LCN-2 KO animals. (A) BALF IL-10 protein levels at 3 days following final DE exposure; (B) BALF CXCL1 levels at 3 days following final DE challenge; (C) percent macrophages, (D) neutrophils, and (E) lymphocytes in BALF by differential cell counting at 3 days following final DE exposure; histopathological images (F) and scoring quantification of ECM deposition (G) and lymphoid aggregates (H) following a 3-day recovery period after final DE instillation. *P ≤ 0.05; ** P < 0.01; based on 2-way ANOVA with Tukey Method for post-hoc comparisons for A-E; based on Kruskal-Wallis tests with Benjamini, Krieger and Yekutieli FDR correction for multiple comparisons. Each symbol indicates a single animal.

Given the recognized role for IL-10 signaling in anti-inflammatory and pro-repair/recovery processes, as well as the inhibition of fibrotic processes [22,23], we chose to explore this pathway as a means of rescuing the negative effects of LCN-2 deficiency. To test this hypothesis, we administered recombinant mouse IL-10 during the recovery period of a repetitive exposure + 3-day recovery study in LCN-2 KO mice (see treatment scheme in Fig 2). To assess the effects of IL-10 administration *in vivo*, we first measured IL-10 protein levels in BALF via ELISA. In DE-exposed LCN-2 KO mice with and without rIL-10 administration, IL-10 was significantly decreased when compared to the saline-treated mice that received the rIL-10 administration (Fig 8A), where 2-way ANOVA analysis identified a main effect of DE exposure on IL-10 levels.

When assessing pro-inflammatory vs. pro-recovery outcomes in LCN-2 KO mice, rIL-10 administration did not alter CXCL1 protein levels (Fig 8B) and macrophage levels remained significantly decreased regardless of rIL-10 treatment (Fig 8C). However, while untreated LCN-2 KO mice exhibited significant upregulation in BALF neutrophils at this 3-day recovery post-DE timepoint, mice administered rIL-10 did not exhibit this increase (Fig 8D). When assessing lymphocyte populations, rIL-10 administration did not alter the DE-mediated increase in these cells (Fig 8E).

To evaluate the impacts of rIL-10 administration on ECM deposition and lymphoid aggregate tissue histopathology parameters, analysis was performed using a Kruskal Wallis test. A significant main effect of rIL-10 administration was observed for both scoring parameters, although post-hoc pairwise comparisons with FDR correction did not reach the q = 0.05 threashold for significance for any two groups (Fig 8F-8H). Overall, these findings indicate that while LCN-2 KO mice exhibit impaired reparative processes in the lung following DE exposure, these deficits are partially rescued by exogenous rIL-10 administration.

An nCounter Fibrosis V2 NanoString panel was used to explore gene transcripts altered by rIL-10 treatment in LCN-2 knockout mice during the recovery period following lung inflammation induced by DE. Notably and consistent with the histopathological findings, numerous genes relating to ECM remodeling were nominally significant when comparing DE-exposed mice treated with rIL-10 to those exposed to DE alone (Table 1). When these genes were input into the String Database, the top biological process by gene ontology was ‘calcium-independent cell-matrix adhesion’ (GO:007161; String Database false discovery rate of 0.00081), while the top Reactome pathway identified was ‘fibronectin matrix formation’ (MMU-1566977; String Database false discovery rate of 0.00093). Together, the deficits in repair/recovery and heightened baseline inflammatory pathology in LCN-2 KO animals imply that LCN-2 plays a role in regulating tissue homeostasis and repair/recovery under inflammatory stimulation in the lung, and that induction of IL-10 signaling can partially rescue this phenotype.

**Table 1:** Gene Transcripts in nCounter Fibrosis V2 Nanostring panel found to be significantly altered between DE-exposed LCN-2 KO animals with vs. without rIL-10 administration.

Gene Name	Fold Regulation	P Value	Associated Process(es)
***Upregulated***			
Fn1	1.22	0.023	ECM Synthesis, EMT, Focal Adhesion Kinase, PI3K-Akt, Platelet Degranulation
Daxx	1.26	0.030	MAPK Cell Stress, Programmed Cell Death
***Downregulated***			
P4hb	1.15	0.002	Collagen Biosynthesis & Modification, Oxidative Stress, Proteotoxic Stress
Postn	1.23	0.002	EMT, Myofibroblast Regulation
Hikeshi	1.53	0.005	Proteotoxic Stress
Dll1	1.70	0.005	Notch, Th1 Differentiation
Itgb1	1.16	0.008	ECM Synthesis, EMT, Focal Adhesion Kinase, PI3K-Akt
Cdh5	1.32	0.011	Angiogenesis
Pdgfb	1.30	0.012	EMT, MAPK Cell Stress, PDGF Signaling, PI3K-Akt, Platelet Degranulation
Hdc	1.34	0.014	Neurotransmission, inflammation, gastric acid secretion, smooth muscle tone
App	1.14	0.020	Adenosine Pathway, ECM Synthesis, Endotoxin Response, Inflammasome, Platelet Degranulation, TLR Signaling
Rac1	1.21	0.023	Angiogenesis, EMT, Granulocyte Activity, MAPK Cell Stress, Neutrophil Degranulation, PI3K-Akt, Phagocytic Cell Function, TLR Signaling, Wnt
Flt4	1.17	0.027	MAPK Cell Stress, PI3K-Akt
Pecam1	1.31	0.028	Neutrophil Degranulation, Platelet Degranulation
Xaf1	1.27	0.029	Type I Interferon
Psmc3	1.17	0.033	Cell Cycle, Granulocyte Activity, Hypoxia, Neutrophil Degranulation
Creb1	1.19	0.033	Adenosine Pathway, Endotoxin Response, Insulin Resistance, PI3K-Akt, TLR Signaling
Ywhaq	1.19	0.034	Cell Cycle, De Novo Lipogenesis, PI3K-Akt
Calm1	1.34	0.034	Adenosine Pathway, Angiogenesis, Granulocyte Activity, Platelet Degranulation
Src	1.18	0.035	Angiogenesis, EMT, Focal Adhesion Kinase, PDGF Signaling
Bcap31	1.07	0.040	Proteotoxic Stress
Kras	1.33	0.044	Angiogenesis, Autophagy, EMT, Granulocyte Activity, Insulin Signaling, MAPK Cell Stress, PDGF Signaling, PI3K-Akt, Programmed Cell Death, mTOR
Tek	1.28	0.046	MAPK Cell Stress, PI3K-Akt

## Discussion

Byproducts of the agricultural industry such as harmful dusts, vapors, and fumes can cause serious respiratory diseases including asthma, interstitial lung disease, COPD, and lung cancer [1]. Some of these harmful substances include but are not limited to dust, pesticides, fertilizers, fumes, and animal wastes. COPD is a leading cause of death worldwide and is most commonly caused by smoking. However, there is growing concern for individuals in the agricultural industry as they are more susceptible to acquiring COPD. This susceptibility to respiratory conditions results in part from the frequent exposure to harmful particulate matter, and it is estimated that 8−32% of agricultural industry workers develop COPD in comparison to 4% of non-farming workers [24]. Thus, it is crucial to gain a better understanding of the molecular mechanisms involved in the progression and resolution of chronic lung inflammation caused by organic dusts. The acute phase protein LCN-2 has pleiotropic roles in host immunity, inflammation, and repair processes, while also being identified as a potential BALF biomarker for patients with COPD [25]. Yet, the role of LCN-2 in the lung remains relatively unexplored [6]. Here, we aimed to determine the role of LCN-2 under homeostatic conditions and in the context of lung injury driven by chronic organic dust inhalation, using a murine model of repetitive DE exposure.

Through these investigations, we identified a striking increase in lung LCN-2 levels in response to DE. Furthermore, we found that LCN-2 was necessary for lung recovery following DE exposure, evidenced by LCN-2 KO mice having enhanced histopathological injury (Fig 4), and an increase in ECM synthesis and degradation pathways activated after repetitive exposure to DE as shown by transcript-level analyses (Fig 7). The lung ECM is composed of proteins that are essential for normal lung development and organ health, providing structural integrity, shaping cell behavior, and acting as a reservoir for cytokines and growth factors [26]. In addition to serving as a physical scaffold for cellular constituents, it initiates critical cues that are required for tissue homeostasis, morphogenesis, and differentiation [27]. ECM synthesis is a coordinated process that is critical in balancing the unique ECM environment via the production of new ECM molecules by surrounding cells. ECM turnover via ECM degradation also plays a crucial role in regulating the ECM environment. In our investigations, we identified in LCN-2 KO mice significant alterations in genes involved with both ECM synthesis and degradation in comparison to WT mice. Our NanoString findings imply that LCN-2 plays a critical role in regulating the repair process in the lung after exposure to DE via modulating ECM synthesis, deposition, and degradation processes that are important for injury response and repair processes. Of note, we also observed increased CXCL1 in DE-exposed LCN-2 KO animals, and CXCL1 has been reported to be regulated by ECM [28,29].

The NanoString findings of altered ECM-related pathways are supported by the prolonged tissue damage observed in the LCN-2 KO mice, which showed deficits in recovery with enhanced histopathology particularly with lymphoid aggregate persistence. The high LCN-2 level in the lungs during basal conditions as compared to serum also suggests an important role of LCN-2 in regulating lung homeostasis in the absence of an insult, and higher baseline inflammatory scoring in LCN-2 KO mice support this concept. It’s important to note that there are no literature exploring a role for LCN-2 in lung development. Thus, it is not known whether the altered LCN-2 baseline histopathology is a result of permanent structural or developmental alterations that could predispose the lungs to an inflammatory state, vs. changes overtime to either the saline instillations, or other inhaled particulate from bedding or other sources. Studies are warranted to investigate these different possibilities.

With regard to the concept of LCN-2 having a role in tissue homeostasis in the context of inflammatory challenges, recent findings of LCN-2 highlight its pleiotropic actions as not only being pro-inflammatory, but in regulating repair and anti-inflammatory processes as well. For example, LCN-2 has been shown to play an important role in dampening levels of pro-inflammatory cytokines and chemokines in the brain in response to inflammation [30,31], thus it is possible that in other tissues such as the lung, LCN-2 regulates inflammation physiology and/or recruitment of immune cells as a protective mechanism. It has also been shown by others that LCN-2 is an important regulator of iron metabolism and iron homeostasis [32] and that iron can regulate both innate and adaptive immunity [33]. It is possible that the increased cellular infiltration observed in the LCN-2 KO mice is due to a disturbance in iron regulation, and this is consequently leading to the recruitment of immune cells to help produce proteins that help regulate iron levels. This concept is supported by the recognized role of disrupted iron homeostasis in various acute and chronic (including infectious and noninfectious) lung diseases [34].

Furthermore, the enhanced histopathology observed with lymphoid aggregate formation and ECM deposition in LCN-2 KO mice in our model lend support to the hypothesis that LCN-2 is involved in the repair process in the lungs. Lymphoid aggregates, also known as secondary lymphoid tissues or inducible bronchus-associated lymphoid tissue (iBALT), that develop in response to DE, have previously been shown to increase with inflammation [35]. In WT mice in our model, these aggregates start to disappear during the recovery period. Other studies using the same *in vivo* DE-exposure model show that the aggregates primarily consist of T cells and B cells, but also contain other immune cell populations [18]. Future characterization of these aggregates via immunolabeling or flow cytometry may help us understand how LCN-2 is interacting with these different immune cells in the lymphoid aggregates to regulate their development and persistence. Taken together, these results in combination with our NanoString findings show a role for LCN-2 in regulating repair of damaged lung tissue induced by repetitive DE challenge.

An unexpected finding in our investigations was the observed compartmentalized effects of LCN-2 in airway versus tissue lung compartments. In lung tissue, LCN-2 KO mice instilled with DE had enhanced levels of alveolar and interstitial macrophages with positive staining for M2-like macrophage markers (CD86 = M1-like; CD206 = M2-like; CD86^-^CD206^+^). Contrary to what we expected, LCN-2 KO mice had higher levels of alveolar and interstitial M2-like macrophages in comparison to WT mice. Elevated levels of CXCL1 protein in mice lacking LCN-2 may contribute to this M2-like macrophage polarization that could result in a non-resolving and aberrant fibroproliferative repair phenotype in these mice [36]. Of note, genotype and recovery period both impacted MARCO (macrophage receptor with collagenous structure) transcript levels, a protein associated with pathogen clearance by macrophages. In contrast to macrophages, we found T cells elevated in BALF but not in the lung homogenates of LCN-2 KO mice. Both macrophage and T cell populations have recognized roles in the lung response to repetitive DE exposure, including evidence of pro-reparative functions [15,37,38]. Based on these alterations in cell populations that regulate anti-inflammatory pathways and altered tissue pathology in DE-exposed LCN-2 KO mice, along with observed detriments in tissue recovery following exposure, we chose to further investigate anti-inflammatory signaling mechanisms in our model. For that purpose, we attempted to rescue the LCN-2 KO-associated defects via exogenous IL-10 administration, given its well-known role in anti-inflammatory signaling and tissue repair [39,40]. Indeed, our data suggest that the crosstalk of LCN-2 and IL-10 is important for recovery in this repetitive DE model, as we found evidence of reduced inflammation seen in recovery when LCN-2 KO mice were administered recombinant IL-10. These findings support the hypothesis that IL-10 mediates tissue repair in this model, and that LCN-2 may be necessary to trigger the appropriate induction and regulation of IL-10. It is also important to note that we observed a decrease in IL-10 protein measured in BALF of LCN-2 KO animals, and this was also observed in rIL-10-administered animals. This implies a possible increase in the degradation, utilization, or clearance of IL-10 protein. Using the NanoString Fibrosis V2 panel, we were also able to identify genes most affected by IL-10 administration in DE-exposed mice during the recovery period. Here, numerous genes were identified that were related to ECM remodeling, and pathway analyses further supported these findings. Given the deficits in resolution of histopathological markers of injury in LCN-2 KO mice, and the partial rescue of these deficits in ECM deposition and lymphoid aggregates in rIL-10-treated mice (where Kruskall-Wallis identified significant variance, although no individual pairwise comparison remained significant after FDR correction for multiple testing), these NanoString analyses further support a role for LCN-2 in regulating repair pathways via IL-10. Overall, these findings support our hypothesis of LCN-2 playing a protective role against lung injury induced by DE, in part by impacting IL-10-mediated anti-inflammatory and pro-repair programs to promote recovery after repetitive DE exposure.

There are numerous limitations associated with these studies that should be carefully considered. Firstly it is not yet clear why we seemingly paradoxically observed increased M2-like macrophages but decreased IL-10 in the LCN-2 KO animals. These data suggest that LCN2 could be required for the production of IL-10 following DE exposure, but IL-10 is also a recognized driver of M2-like (and particularly “M2c”) macrophage polarization [41]. Although, there are other known drivers of this polarization, including IL-4, and future studies to decipher how LCN-2 impacts polarization within the umbrella alternatively activated macrophage phenotype is warranted. Additionally, our findings of an LCN-2/IL-10 axis are largely associative at this point, with only partial rescue of the LCN-2 KO phenotype with rIL-10 administration. Future mechanistic studies are warranted to establish the interplay of IL-10 and LCN-2 in this context. In addition, we recognize that providing the rIL-10 administration on the last day of the final DE dose makes it difficult to distinguish altered active healing of existing damage from reduced overall inflammation severity from the final DE challenge. Although, we have previously reported temporal recovery for up to 4 weeks following final DE [37]. Interestingly, the lymphoid aggregates that we observe only occur with repetitive DE challenges over time (they do not develop with a single DE challenge) and, once developed, these aggregates persist the longest as compared to the other markers of inflammatory pathology associated with the DE exposure model [37]. Therefore, we hypothesize that the rIL-10-associated partial rescue is due to active healing of existing damage, although our observed reduction in neutrophilia could be associated with a reduction in the severity of the final inflammatory insult. Furthermore, our Nanostring gene expression analyses were performed with a limited number of samples (N = 3 per group), limiting our capacity to perform subsequent analyses to account for false discovery rate. These studies should thus be interpreted conservatively, although they are corroborated by our *in vivo* histopathological findings. Finally, it is possible that saline instillation followed by BALF collection and lung inflation for formalin fixation and paraffin embedding could have led to alterations in histopathology between WT and LCN2 control animals, future studies with mice not given saline or lavaged would minimize potential artifacts and/or impacts of these techniques. Ultimately, follow-up studies are warranted to validate our initial findings presented in this manuscript.

## Conclusion

Overall, our findings highlight a protective role of LCN-2 in lung homeostasis as well as in the context of inflammation associated with DE exposure. Further studies to explore the connection between LCN-2 and IL-10 signaling to promote lung recovery following environmental dust-induced injury are warranted. Such investigations will be important in elucidating specific mechanisms involved with the repair process resolving injury brought about by environmental dust exposures, and would support feasibility studies aimed at assessing the therapeutic utility of LCN-2 in the future in patients with chronic lung conditions.

## Supporting information

S1 Fig Gating Strategy (A) and Population Overview (B) of BALF Samples used for Flow Cytometry. (A) Forward (FSC-A) vs Side Scatter (SSC-A) were used to gate out live cells, followed by a Live/Dead Stain vs SSC-A, and FSC-A vs FSC-H to gate out single cells. From there, different combinations of markers were used to gate out specific immune cells as shown below. (B) Immune cell populations are highlighted within entire cell population used for analysis per group.
(PNG)

S2 Fig Serum levels of LCN-2 in WT and LCN2 KO animals following repetitive DE or saline exposure. Serum LCN-2 protein levels in WT and and LCN-2 KO mice at 5 hours following final DE exposure. Assay confirms lack of LCN-2 protein in KO mice. *P < 0.05 based on Unpaired t test between WT saline and DE-exposed mice. Each symbol represents a single animal.
(PNG)

S3 Fig RNAscope representative images of mouse lungs stained for LCN-2 mRNA (red). Three ranges of LCN-2 mRNA fluorescence signal are shown for two WT DE mouse lungs. Note the wide range of fluorescence intensities for the LCN-2 mRNA signal. The green box indicates the fluorescence intensity range chosen for quantification of LCN-2 mRNA. Mouse WT DE samples chosen to select range was based off of samples showing LCN-2 mRNA signal on the lower and higher cutoffs. Scale bars in gray in lower left of each image represent 10 µm; gradient keys on upper right of each image indicate expression gradient for LCN-2 (red) and DAPI (blue).
(PNG)

## References

[1] Nordgren TM, Bailey KL. Pulmonary health effects of agriculture. Curr Opin Pulm Med. 2016;22(2):144–9. doi: 10.1097/MCP.0000000000000247 26761627 PMC4764055

[2] Bailey KL, Meza JL, Smith LM, Von Essen SG, Romberger DJ. Agricultural exposures in patients with COPD in health systems serving rural areas. J Agromedicine. 2007;12(3):71–6. doi: 10.1300/J096v12n02_10 19042672

[3] Cao Y, Bender IK, Konstantinidis AK, Shin SC, Jewell CM, Cidlowski JA, et al. Glucocorticoid receptor translational isoforms underlie maturational stage-specific glucocorticoid sensitivities of dendritic cells in mice and humans. Blood. 2013;121(9):1553–62. doi: 10.1182/blood-2012-05-432336 23297131 PMC3587320

[4] La Manna G, Ghinatti G, Tazzari PL, Alviano F, Ricci F, Capelli I, et al. Neutrophil gelatinase-associated lipocalin increases HLA-G(+)/FoxP3(+) T-regulatory cell population in an in vitro model of PBMC. PLoS One. 2014;9(2):e89497. doi: 10.1371/journal.pone.0089497 24586826 PMC3937322

[5] Warszawska JM, Gawish R, Sharif O, Sigel S, Doninger B, Lakovits K, et al. Lipocalin 2 deactivates macrophages and worsens pneumococcal pneumonia outcomes. J Clin Invest. 2013;123(8):3363–72. doi: 10.1172/JCI67911 23863624 PMC3726165

[6] Guardado S, Ojeda-Juárez D, Kaul M, Nordgren TM. Comprehensive review of lipocalin 2-mediated effects in lung inflammation. Am J Physiol Lung Cell Mol Physiol. 2021;321(4):L726–33. doi: 10.1152/ajplung.00080.2021 34468208 PMC8560400

[7] Xiao Y, Yan W, Zhou K, Cao Y, Cai W. Glucocorticoid treatment alters systemic bile acid homeostasis by regulating the biosynthesis and transport of bile salts. Dig Liver Dis. 2016;48(7):771–9. doi: 10.1016/j.dld.2016.03.022 27133208

[8] Schroll A, Eller K, Feistritzer C, Nairz M, Sonnweber T, Moser PA, et al. Lipocalin-2 ameliorates granulocyte functionality. Eur J Immunol. 2012;42(12):3346–57. doi: 10.1002/eji.201142351 22965758

[9] Boissy RJ, Romberger DJ, Roughead WA, Weissenburger-Moser L, Poole JA, LeVan TD. Shotgun pyrosequencing metagenomic analyses of dusts from swine confinement and grain facilities. PLoS One. 2014;9(4):e95578. doi: 10.1371/journal.pone.0095578 24748147 PMC3991671

[10] Dominguez EC, Phandthong R, Nguyen M, Ulu A, Guardado S, Sveiven S, et al. Aspirin-Triggered resolvin D1 reduces chronic dust-induced lung pathology without altering susceptibility to dust-enhanced carcinogenesis. Cancers (Basel). 2022;14(8):1900. doi: 10.3390/cancers14081900 35454807 PMC9032113

[11] Bachman MA, Miller VL, Weiser JN. Mucosal lipocalin 2 has pro-inflammatory and iron-sequestering effects in response to bacterial enterobactin. PLoS Pathog. 2009;5(10):e1000622. doi: 10.1371/journal.ppat.1000622 19834550 PMC2757716

[12] Bachman MA, Lenio S, Schmidt L, Oyler JE, Weiser JN. Interaction of lipocalin 2, transferrin, and siderophores determines the replicative niche of Klebsiella pneumoniae during pneumonia. mBio. 2012;3(6):e00224-11. doi: 10.1128/mBio.00224-11 23169997 PMC3509427

[13] Wang X, Zhang C, Zou N, Chen Q, Wang C, Zhou X, et al. Lipocalin-2 silencing suppresses inflammation and oxidative stress of acute respiratory distress syndrome by ferroptosis via inhibition of MAPK/ERK pathway in neonatal mice. Bioengineered. 2022;13(1):508–20. doi: 10.1080/21655979.2021.2009970 34969358 PMC8805876

[14] Ulu A, Velazquez JV, Burr A, Sveiven SN, Yang J, Bravo C, et al. Sex-specific differences in resolution of airway inflammation in. Front Pharmacology. 2021;12:785193. doi: 10.3389/fphar.2021.785193 35095496 PMC8793679

[15] Ulu A, Sveiven S, Bilg A, Velazquez JV, Diaz M, Mukherjee M, et al. IL-22 regulates inflammatory responses to agricultural dust-induced airway inflammation. Toxicol Appl Pharmacol. 2022;446:116044. doi: 10.1016/j.taap.2022.116044 35525330 PMC9133182

[16] Poole JA, Alexis NE, Parks C, MacInnes AK, Gentry-Nielsen MJ, Fey PD, et al. Repetitive organic dust exposure in vitro impairs macrophage differentiation and function. J Allergy Clin Immunol. 2008;122(2):375–82, 382.e1-4. doi: 10.1016/j.jaci.2008.05.023 18585769 PMC2685162

[17] Nordgren TM, Bauer CD, Heires AJ, Poole JA, Wyatt TA, West WW, et al. Maresin-1 reduces airway inflammation associated with acute and repetitive exposures to organic dust. Transl Res. 2015;166(1):57–69. doi: 10.1016/j.trsl.2015.01.001 25655838 PMC4458456

[18] Poole JA, Wyatt TA, Oldenburg PJ, Elliott MK, West WW, Sisson JH, et al. Intranasal organic dust exposure-induced airway adaptation response marked by persistent lung inflammation and pathology in mice. Am J Physiol Lung Cell Mol Physiol. 2009;296(6):L1085-95. doi: 10.1152/ajplung.90622.2008 19395665 PMC2692812

[19] Romberger DJ, Bodlak V, Von Essen SG, Mathisen T, Wyatt TA. Hog barn dust extract stimulates IL-8 and IL-6 release in human bronchial epithelial cells via PKC activation. J Appl Physiol (1985). 2002;93(1):289–96. doi: 10.1152/japplphysiol.00815.2001 12070216

[20] Szklarczyk D, Gable AL, Nastou KC, Lyon D, Kirsch R, Pyysalo S, et al. The STRING database in 2021: customizable protein-protein networks, and functional characterization of user-uploaded gene/measurement sets. Nucleic Acids Res. 2021;49(D1):D605–12. doi: 10.1093/nar/gkaa1074 33237311 PMC7779004

[21] Wyatt TA, Nemecek M, Chandra D, DeVasure JM, Nelson AJ, Romberger DJ, et al. Organic dust-induced lung injury and repair: Bi-directional regulation by TNFα and IL-10. J Immunotoxicol. 2020;17(1):153–62. doi: 10.1080/1547691X.2020.1776428 32634062 PMC11238278

[22] Saraiva M, Vieira P, O’Garra A. Biology and therapeutic potential of interleukin-10. J Exp Med. 2020;217(1):e20190418. doi: 10.1084/jem.20190418 31611251 PMC7037253

[23] Poole JA, Nordgren TM, Heires AJ, Nelson AJ, Katafiasz D, Bailey KL, et al. Amphiregulin modulates murine lung recovery and fibroblast function following exposure to agriculture organic dust. Am J Physiol Lung Cell Mol Physiol. 2020;318(1):L180–91. doi: 10.1152/ajplung.00039.2019 31693392 PMC6985879

[24] Bailey KL, Meza JL, Smith LM, Von Essen SG, Romberger DJ. Agricultural exposures in patients with COPD in health systems serving rural areas. J Agromedicine. 2007;12(3):71–6. doi: 10.1300/J096v12n02_10 19042672

[25] Treekitkarnmongkol W, Hassane M, Sinjab A, Chang K, Hara K, Rahal Z. Augmented lipocalin-2 is associated with chronic obstructive pulmonary disease and counteracts lung adenocarcinoma development. American Journal of Respiratory and Critical Care Medicine. 2021;203(1). doi: 10.1164/rccm.202004-1079OC 32730093 PMC7781147

[26] Hackett TL, Osei ET. Modeling extracellular matrix-cell interactions in lung repair and chronic disease. Cells. 2021;10(8):Epub 20210820. doi: 10.3390/cells10082145 34440917 PMC8394761

[27] Frantz C, Stewart KM, Weaver VM. The extracellular matrix at a glance. J Cell Sci. 2010;123(Pt 24):4195–200. doi: 10.1242/jcs.023820 21123617 PMC2995612

[28] Ribaux P, Ehses JA, Lin-Marq N, Carrozzino F, Böni-Schnetzler M, Hammar E, et al. Induction of CXCL1 by extracellular matrix and autocrine enhancement by interleukin-1 in rat pancreatic beta-cells. Endocrinology. 2007;148(11):5582–90. doi: 10.1210/en.2007-0325 17702850

[29] Liu G, Cooley MA, Nair PM, Donovan C, Hsu AC, Jarnicki AG, et al. Airway remodelling and inflammation in asthma are dependent on the extracellular matrix protein fibulin-1c. J Pathol. 2017;243(4):510–23. doi: 10.1002/path.4979 28862768

[30] Ojeda-Juárez D, Shah R, Fields JA, Harahap-Carrillo IS, Koury J, Maung R, et al. Lipocalin-2 mediates HIV-1 induced neuronal injury and behavioral deficits by overriding CCR5-dependent protection. Brain Behav Immun. 2020;89:184–99. doi: 10.1016/j.bbi.2020.06.016 32534984 PMC8153086

[31] Kang SS, Ren Y, Liu C-C, Kurti A, Baker KE, Bu G, et al. Lipocalin-2 protects the brain during inflammatory conditions. Mol Psychiatry. 2018;23(2):344–50. doi: 10.1038/mp.2016.243 28070126 PMC5503822

[32] Neves J, Haider T, Gassmann M, Muckenthaler MU. Iron homeostasis in the lungs-A balance between health and disease. Pharmaceuticals (Basel). 2019;12(1):5. doi: 10.3390/ph12010005 30609678 PMC6469191

[33] Ni S, Yuan Y, Kuang Y, Li X. Iron metabolism and immune regulation. Front Immunol. 2022;13:816282. doi: 10.3389/fimmu.2022.816282 35401569 PMC8983924

[34] Ghio AJ. Disruption of iron homeostasis and lung disease. Biochim Biophys Acta. 2009;1790(7):731–9. doi: 10.1016/j.bbagen.2008.11.004 19100311

[35] Wyatt TA, Nemecek M, Chandra D, DeVasure JM, Nelson AJ, Romberger DJ, et al. Organic dust-induced lung injury and repair: Bi-directional regulation by TNFα and IL-10. J Immunotoxicol. 2020;17(1):153–62. doi: 10.1080/1547691X.2020.1776428 32634062 PMC11238278

[36] Huang W, Jiang M, Lin Y, Qi Y, Li B. Crosstalk between cancer cells and macrophages promotes OSCC cell migration and invasion through a CXCL1/EGF positive feedback loop. Discov Oncol. 2024;15(1):145. doi: 10.1007/s12672-024-00972-8 38713320 PMC11076430

[37] Warren KJ, Wyatt TA, Romberger DJ, Ailts I, West WW, Nelson AJ, et al. Post-injury and resolution response to repetitive inhalation exposure to agricultural organic dust in mice. Safety (Basel). 2017;3(1):10. doi: 10.3390/safety3010010 29387711 PMC5788309

[38] Ulu A, Burr A, Heires AJ, Pavlik J, Larsen T, Perez PA, et al. A high docosahexaenoic acid diet alters lung inflammation and recovery following repetitive exposure to aqueous organic dust extracts. J Nutr Biochem. 2021;97:108797. doi: 10.1016/j.jnutbio.2021.108797 34126202 PMC8725620

[39] Chai Y-S, Chen Y-Q, Lin S-H, Xie K, Wang C-J, Yang Y-Z, et al. Curcumin regulates the differentiation of naïve CD4+T cells and activates IL-10 immune modulation against acute lung injury in mice. Biomed Pharmacother. 2020;125:109946. doi: 10.1016/j.biopha.2020.109946 32004976

[40] Zhang Z-T, Zhang D-Y, Xie K, Wang C-J, Xu F. Luteolin activates Tregs to promote IL-10 expression and alleviating caspase-11-dependent pyroptosis in sepsis-induced lung injury. Int Immunopharmacol. 2021;99:107914. doi: 10.1016/j.intimp.2021.107914 34246059

[41] Lurier EB, Dalton D, Dampier W, Raman P, Nassiri S, Ferraro NM, et al. Transcriptome analysis of IL-10-stimulated (M2c) macrophages by next-generation sequencing. Immunobiology. 2017;222(7):847–56. doi: 10.1016/j.imbio.2017.02.006 28318799 PMC5719494

